# Association between the overexpression of Her3 and clinical pathology and prognosis of colorectal cancer: A meta-analysis

**DOI:** 10.1097/MD.0000000000012317

**Published:** 2018-09-14

**Authors:** Qingying Yan, Kaibo Guo, Guan Feng, Feiyu Shan, Leitao Sun, Kai Zhang, Fengfei Shen, Minhe Shen, Shanming Ruan

**Affiliations:** a The First Clinical Medical College of Zhejiang Chinese Medical University; b Department of Medical Oncology, The First Affiliated Hospital of Zhejiang Chinese Medical University, Hangzhou; c Department of Traditional Chinese Medicine, the First Affiliated Hospital of Wenzhou Medical University, Wenzhou, Zhejiang, China.

**Keywords:** cetuximab, colorectal cancer, human epidermal growth factor receptor-3, prognosis

## Abstract

**Background:**

This study aimed to investigate the association between the overexpression of human epidermal growth factor receptor-3 (Her3) and the clinicopathological parameters and survival of patients with colorectal cancer (CRC).

**Methods:**

Relevant studies on the overexpression of Her3 (measured by immunohistochemistry) and overall survival (OS) in patients with CRC were searched for in PubMed, EMBASE, and Cochrane Library. Published data were extracted and computed into odds ratios (ORs) for assessing the association of Her3 overexpression with tumor differentiation, tumor node metastasis (TNM) stage, position of colon cancer, sex, and age. Prognostic data were computed into hazard ratios (HRs) for OS.

**Results:**

Eight studies including 1716 patients with CRC were included in this meta-analysis. The results revealed a significant association between Her3 overexpression and tumor differentiation [OR = 2.38; 95% confidence interval (95% CI): 1.76–3.22; *P* < .001], TNM tumor stage (OR = 0.71; 95% CI: 0.53–0.96; *P* = .03), and position of colon cancer (OR = 1.71; 95% CI: 1.28–2.27; *P* < .001). While patients with Her3 overexpression demonstrated a worse tumor response (OR = 0.31; 95% CI: 0.16–0.60; *P* < .001) and OS after treatment with cetuximab (HR = 1.86; 95% CI: 1.24–2.79; *P* = .003), they demonstrated better OS after symptomatic treatment (HR = 0.65; 95% CI: 0.50–0.85; *P* = .002). Her3 overexpression was not associated with sex (OR = 1.03; 95% CI: 0.83–1.28; *P* = .79), age (OR = 0.96; 95% CI: 0.75–1.24; *P* = .77), colon or rectum site (OR = 0.79; 95% CI: 0.44–1.43; *P* = .44), and total OS (HR = 1.09; 95% CI: 0.69–1.72; *P* = .72).

**Conclusion:**

Her3 expression is associated with the clinical pathology and prognosis of CRC, which explains the nonefficacy of cetuximab treatment in patients with CRC.

## 1 Introduction

Colorectal cancer (CRC) is one of the leading causes of cancer-related deaths, and its incidence has been increasing in China.^[1,2]^ Some risk factors for CRC include diet, obesity, smoking, inflammatory bowel disease, and lack of physical activity.^[3,4]^ The development of targeted anticancer agents, such as cetuximab and panitumumab, in the last few decades has effectively prolonged the survival rate of patients with CRC.^[5]^ However, the survival rate and prognosis of patients with CRC are not ideal. Thus, it is essential to explore a novel biomarker for a more precise treatment of CRC.

The human epidermal growth factor receptor (Her or ErbB) family of receptor tyrosine kinases, which often expresses, amplifies, or mutates in various cancers,^[6]^ constitutes an essential therapeutic target for the disease. Several cancers, particularly breast, ovarian, and nonsmall cell lung cancers, present overexpression and mutation in ErbB receptors.^[7]^ Her3 is a member of the Her complex (EGFR/HER1/ErbB1, HER2/ErbB2, HER3/ErbB3, and HER4/ErbB4) and plays an essential role in cell proliferation and survival.^[8]^ The activation of Her3 is dependent on binding to and/or heterodimerization with other ErbB receptors,^[9]^ which plays a critical role in the regulation of the Her signaling cascade. In CRC cells, Her3 plays a role in promoting cell proliferation and mediating cell-to-chemotherapy and targeted drug resistance, thereby promoting the invasion and migration of CRC. Previous research has reported that cetuximab improves AZD6244 antitumor activity in CRC HT29 cells in vitro and in nude mice by attenuating Her3/Akt pathway activation.^[10]^ In vitro, ERBB3 knockdown decreases cell proliferation, induces apoptosis, and blocks the migration of colon cancer cells.^[11]^ At the same time, in BRAF-V600E mutant colon cancer stem cells (CSCs), HER3/Neuregulin-1β induces cellular proliferation and drug resistance to vemurafenib.^[12]^

Her3 has garnered substantial attention, and anti-Her3 antibodies have been developed for therapeutic use. Patritumab, a Her3-targeted antibody, has been used for treating nonsmall cell lung cancer,^[13]^ the efficacy of which is enhanced when used in combination with erlotinib. Some studies have highlighted an association between Her3 overexpression and metastases in CRC, and Her3 has been identified as a predictive factor for clinical outcome in patients with wild-type KRAS CRC treated with cetuximab.^[14–16]^ However, no consensus has been reached on the association between Her3 expression and CRC. For instance, Kountourakis et al ^[17]^ did not find any major association between Her3 expression and tumor biology in CRC. Thus, the association between Her3 overexpression and prognosis in CRC remains controversial.

Hence, this meta-analysis was performed to elucidate the association between Her3 overexpression and clinical pathology and prognosis of CRC. Parameters included tumor differentiation, TNM stage, colon cancer position, sex, age, and prognosis [overall survival (OS)]. The primary aim of this study was to evaluate the Her3 overexpression in CRC, thereby allowing more rational development of therapeutic strategies targeting this receptor. Furthermore, we hope to provide a theoretical basis for the accurate treatment of CRC.

## 2 Methods

### 2.1 Inclusion and exclusion criteria

Inclusion criteria were as follows: pathological diagnosis with CRC; Her3 expression detected in the cytoplasm or membrane and evaluated by immunohistochemistry (IHC); and availability of information on clinicopathological parameters and/or OS. Exclusion criteria were as follows: case reports, meeting, and reviews; evaluation method other than IHC; and duplication. All analyses were based on previous published studies; thus, no ethical approval and patient consent are required.

### 2.2 Search strategy

The PubMed, EMBASE, and Cochrane Library databases updated from January 1990 to March 2017 were searched for studies on the overexpression of Her3 and clinical pathology and prognosis of CRC. The following terms were searched: “colorectal cancer,” “colorectal carcinoma,” “colorectal,” “colon cancer,” “colon carcinoma,” “rectal cancer,” “rectal carcinoma,” “rectum cancer,” “human epidermal growth factor receptor-3,” “her3,” “her-3,” and “c-erbB-3.” The search was restricted to only English studies.

### 2.3 Study selection and data extraction

On the basis of the inclusion criteria, all relevant data, including general information (age, sex, tumor differentiation, and primary site), specific data (Her3 expression status), and survival data, were extracted by 2 independent investigators (Qingying Yan and Kaibo Guo). Disagreements in findings were resolved by a discussion until a consensus was reached.

### 2.4 Risk of bias assessment

Funnel plots were used to assess the publication bias. Furthermore, Begg funnel plot and Egger regression test were used to assess any potential publication bias. *P* > .1 indicated no publication bias.

### 2.5 Statistical analysis

Revman 5.2 (Copenhagen: The Nordic Cochrane Centre, The Cochrane Collaboration) and STATA 12.0 (StataCorp, College Station, Texas) were used for data analysis. Odds ratios (ORs) and 95% confidence intervals (95% CIs) were used to estimate the association between Her3 overexpression and clinicopathological parameters in CRC. OS was reported with a hazard ratio (HR), which was directly pooled from the articles. Engauge Digitizer 4.1 software (http://digitizer.sourceforge.net) was used to calculate HR from the Kaplan–Meier survival curves. The *I*^*2*^ value and *χ*^*2*^ test were used to assess statistical heterogeneity. *P* < .1 or *I*^*2*^ > 50% indicated moderate-to-high heterogeneity. Data were analyzed by the random effects model when statistical heterogeneity was found; otherwise, the fixed effects model was used. All the results were presented as forest plots.

### 2.6 Quality assessment

The Newcastle–Ottawa scale^[18]^ was used for quality assessment by 2 investigators (Qingying Yan and Guan Feng). Disagreements were resolved by a discussion with the third investigator (Feiyu Shan). The scores of included literature were recorded until consistency was achieved.

## 3 Results

### 3.1 Study selection

**Figure 1:**
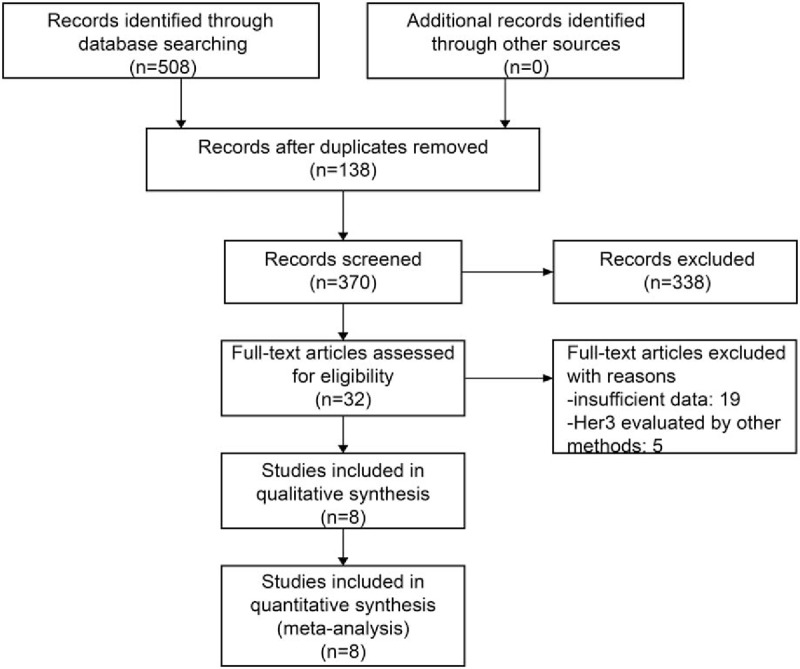
Flowchart for study selection.

Figure 1 shows 508 potentially relevant studies. Of these, 476 articles (338 irrelevant and 138 duplicate articles) were excluded. The remaining 32 articles were screened for titles and abstracts, of which 24 were excluded (19 articles with insufficient data and 5 with methods other than IHC to evaluate the Her3 status). Finally, 8 studies^[14–17,19–22]^ were included in this meta-analysis. In these studies, the proportion of Her3 overexpression ranged from 13.4% to 80.3%, and the proportion of high Her3 expression in all patients was 65.9% (1131/1716). Table 1 summarizes the cut-off for Her3 overexpression and clinicopathological factors. Her3 expression in 6 studies^[15–17,19–20,22]^ was graded as follows: grade 0 to 1 was categorized as low expression and grade 2 to 3 as high expression. Furthermore, 2 studies^[14,21]^ were evaluated according to the Rajkumar score, and the specificity for estimating Her-3 expression was set at ≤8.

**Table 1:**
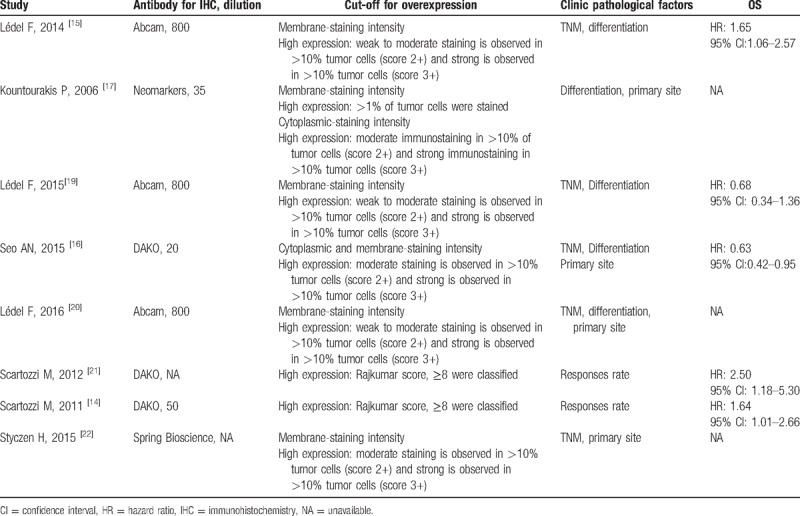
The cut-off for Her3 overexpression and clinic pathological factors.

### 3.2 Study characteristics and quality assessment

Table 2 summarizes the characteristics of the included studies. Overall, 1716 patients with CRC, including 45% males (773/1716) and 55% females (943/1716), were included in the 8 eligible studies. In 1131 (65.9%) patients, Her3 overexpression was detected in the cytoplasm and membrane. The study quality score ranged from 5 to 8, and articles with a score of ≥6 were regarded as having high quality. Among the articles, 5 studies^[12–14,16–17]^ compared the association between Her3 overexpression and tumor differentiation, 4^[15,19,20,22]^ reported the asscociation between high Her3 expression and TNM stage, 4^[16,17,20,22]^ assessed the distribution of Her3 overexpression in left- and right-sided colon cancers, 5^[15,17,19,20,22]^ analyzed the distribution of Her3 overexpression at the site of colon or rectal cancer, 3^[15,16,20]^ compared the difference in Her3 overexpression between patients aged 66 and >66 years, 2^[14,21]^ reported tumor response in case of high and low Her3 expression, 6^[14–16,19,21,22]^ compared OS between patients with high and low Her3 expression, and 3^[15,20,21]^ mentioned HR and 95% CIs. The remaining studies^[14,19,22]^ were assessed using Engauge Digitizer 4.1 through survival curves.

**Table 2:**
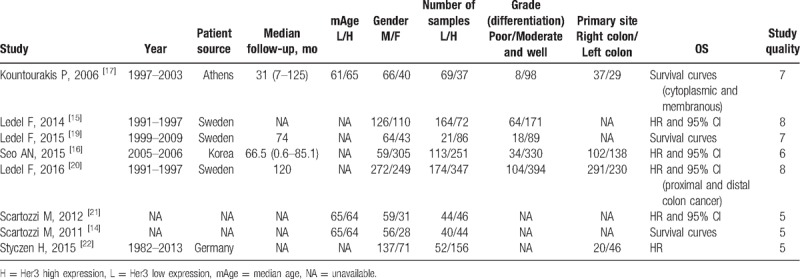
The characteristics of studies.

### 3.3 Correlation between Her3 overexpression and clinicopathological parameters of patients with CRC

**Figure 2:**
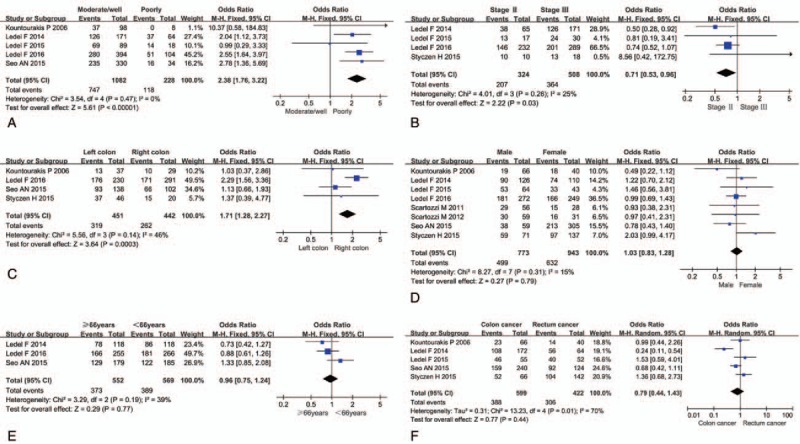
(A) Correlation between high Her3 expression and tumor differentiation. Low-grade, well, and moderately differentiated carcinoma; high-grade, poorly differentiated, and undifferentiated carcinoma. (B) Correlation between high Her3 expression and tumor stage. (C) Correlation between high Her3 expression and left- and right-sided colon cancer. Right cancer, cecum, ascending, transverse colon; left cancer, descending, sigmoid colon. (D) Correlation between high Her3 expression and gender. (E) Correlation between high Her3 expression and the age of 66 years. (F) Correlation between high Her3 expression and colon or rectum cancer.

On assessing the data, a significant association was found between high Her3 expression and tumor differentiation (OR = 2.38; 95% CI: 1.76–3.22; *P* < .001; Fig. 2A), tumor stage (OR = 0.71; 95% CI: 0.53–0.96; *P* = .03; Fig. 2B), and position of colon cancer (OR = 1.71; 95% CI: 1.28–2.27; *P* < .001; Fig. 2C). Her3 overexpression was not associated with gender (OR = 1.03; 95% CI: 0.83–1.28; *P* = .79; Fig. 2D), age (OR = 0.96; 95% CI: 0.75–1.24; *P* = .77; Fig. 2E), and colon or rectum cancer (OR = 0.79; 95% CI: 0.44–1.43; *P* = .44; Fig. 2F).

### 3.4 Correlation between overexpression of Her3 and prognosis of patients with CRC

**Figure 3:**
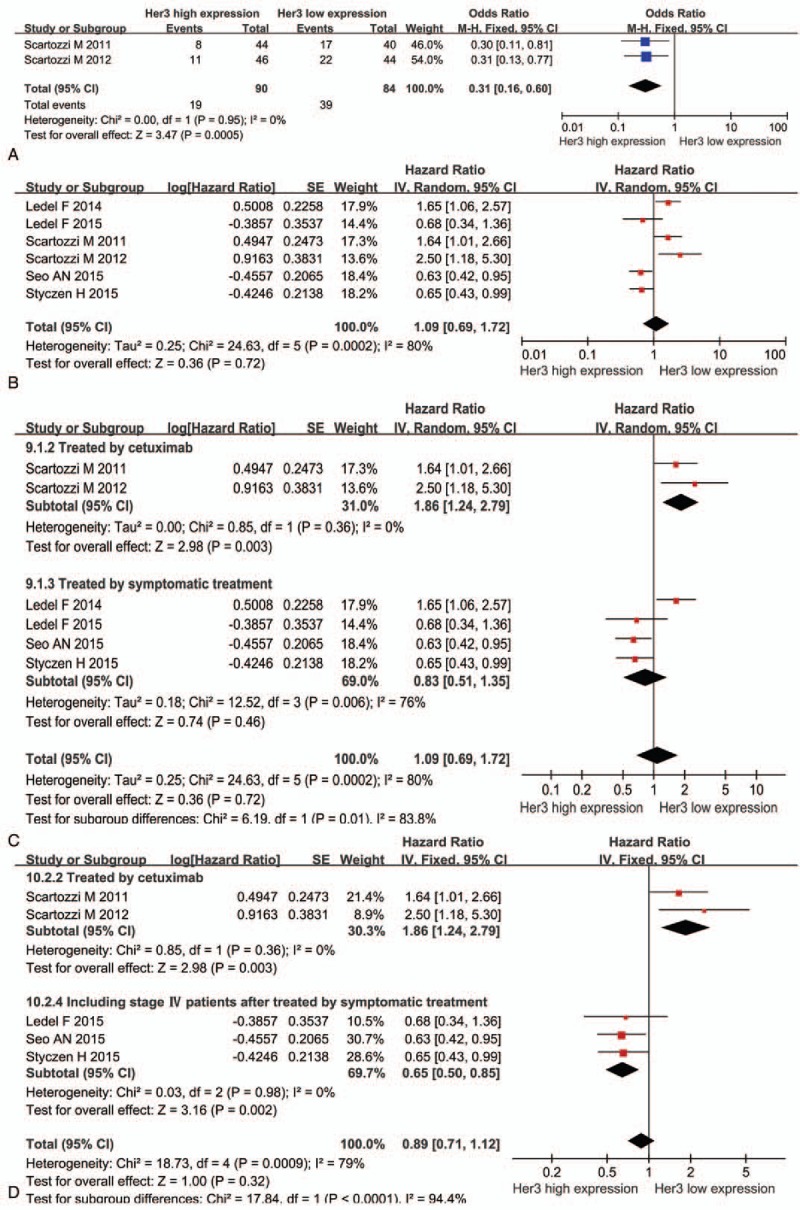
(A) Correlation between Her3 expression and tumor response. (B) Correlation between Her3 expression and OS. (C) Subgroup analysis for Her3 expression and OS. (D) Subgroup analysis after sensitivity analysis.

Two studies elucidated the tumor response between high and low Her3 expression in patients treated with cetuximab-based chemotherapy. High Her3 expression was observed in 90 patients, while low expression was observed in 84. Among patients with high and low expression, partial response was observed in 19 (21.1%) and 39 patients (46.4%), respectively. High Her3 expression had a worse tumor response (OR = 0.31; 95% CI: 0.16–0.60; *P* < .001; Fig. 3A).

Among the 8 articles, 1 study reported OS in patients with a high Her3 expression either in the membrane or cytoplasm and 1 study reported OS in patients with a high Her3 expression in the proximal and distal colon. Synthesis analysis of the remaining 6 studies revealed no association between Her3 overexpression and survival (HR = 1.09; 95% CI: 0.69–1.72; *P* = .72; Fig. 3B).

### 3.5 Subgroup analysis

Notably, significant heterogeneity was present among the studies. Further analysis revealed that high heterogeneity was significantly associated with the research object and treatment. We noticed that among the 6 studies, 2 used cetuximab and cetuximab-based chemotherapy for treating metastatic CRC (mCRC). Furthermore, in the remaining 4 studies, 1 study examined patients with tumor stages II-III treated by symptomatic treatment such as radiotherapy, chemotherapy, or targeted therapy; 1 study investigated patients with stages II–IV CRC; and the remaining 2 studies investigated patients with stages I–IV CRC.

Therefore, a subgroup analysis was conducted based on cetuximab-based therapy or symptomatic treatment (Fig. 3C). As shown in Fig. 3C, patients with low Her3 expression had a better OS after being treated with cetuximab or cetuximab-based chemotherapy (HR = 1.86; 95% CI: 1.24–2.79; *P* = .003); however, we did not find OS improvement in patients with high Her3 expression after symptomatic treatment (HR = 0.83; 95% CI: 0.51–1.35; *P* = .46; Fig. 3C). High heterogeneity also existed in patients after symptomatic treatment, and the TNM stage explained this condition. Ledel et al ^[15]^ reported Her3 overexpression in patients with stages II–III CRC, whereas the remaining 3 articles included patients with stage IV. After sensitivity analysis, the results suggested that patients with high Her3 expression had a better OS after symptomatic treatment (HR = 0.65; 95% CI: 0.50–0.85; *P* = .002; Fig. 3D).

### 3.6 Publication bias

**Figure 4:**
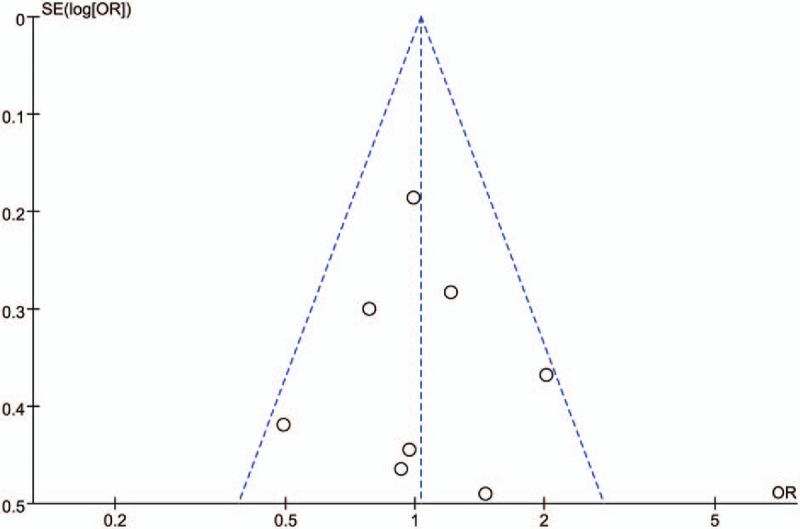
Funnel plot.

The funnel plot of the correlation between Her3 overexpression and OS (Fig. 4) showed that no significant publication bias existed in this research, indicating that the obtained results were relatively reliable. Begg funnel plot and Egger test did not reveal any evidence of obvious asymmetry in the studies. The *P* values in Begg plot and Egger test were 1.0 and 0.978 for tumor differentiation, 0.734 and 0.47 for tumor stage, 1.0 and 0.364 for the position of left- or right-sided colon cancer, 0.805 and 0.902 for gender, 1.0 and 0.9 for age of 66 years, 0.806 and 0.797 for colon or rectum cancer, and 0.133 and 0.435 for OS, respectively.

## 4 Discussion

This meta-analysis suggested that the overexpression of Her3 tends to occur more frequently in moderately/well-differentiated tumors and the left colon in CRC. In addition, stage III disease was positively associated with high Her3 expression compared with stage II disease. Furthermore, it is worth noting that high Her3 expression is related to worse survival in patients receiving cetuximab or cetuximab-based chemotherapy. However, patients with high Her3 expression had better OS with symptomatic treatment. Taken together, Her3 overexpression may have a favorable impact on the prognosis of CRC; however, it may cause failure of cetuximab treatment in these patients.

Her3 overexpression, which is present in various carcinomas, is related to poor prognosis and is involved in the development of resistance to therapy.^[23]^ One study highlighted the close relationship of Her3 with the development of CRC, and approximately 36% to 90% of patients with CRC have expression of Her3,^[24]^ which is often coexpressed with EGFR and Her2. However, a meta-analysis demonstrated that the mortality risk was considerably higher for people who had Her3 overexpression rather than for those with lower expression in solid tumors.^[25]^ The results of the present study corroborate with those of previous studies, suggesting that Her3 overexpression is associated with CRC.

The distinction between the left and right colon has been gaining increasing momentum. Hansen and Jess ^[26]^ performed a meta-analysis in which patients with right-sided colon cancer had a worse prognosis than those with left-sided colon cancer. Petrelli et al^[27]^ suggested that patients with left-sided colon cancer had better OS than those with right-sided colon cancer. Our results revealed that high Her3 expression tends to occur more frequently in patients with left-side colon cancer, which is in line with the results of some previous studies.

Furthermore, Warschkow et al^[28]^ reported that patients with right-sided colon cancer had a better OS in TNM stages I–II, but those with right- and left-sided colon cancer had a similar prognosis for TNM stage III. Weiss et al^[29]^ analyzed 53,801 patients with CRC and showed that patients with stage I cancer had a similar prognosis for both left- and right-sided colon cancers; however, the 5-year mortality rate for patients with left-sided colon cancer was higher in stage II cancer than in III cancer. In population-based patients with stage III-IV CRC, patients with right-sided primary tumors had poorer survival than those with left-sided colon cancer.^[30]^ All these studies demonstrated that the tumor location was related to the TNM tumor stage for the prognosis of CRC.

Over the last few years, surgical resection has been the principal treatment strategy for CRC; nonetheless, not all patients can benefit from surgery. Apparently, chemotherapy resistance and targeted drug resistance are the leading causes of treatment failure in patients with mCRC. Studies have established that the activation of the Her3 receptor signaling pathway is the primary cause of cancer treatment failure and drug resistance. Jacobsen et al^[31]^ and Li et al^[32]^ have found that the expression of Her3 increased in breast cancer resistant cell lines (MDA-MB-175-VII) and ovarian cancer drug-resistant cell lines (SKOV3-T) after trastuzumab treatment. Nakata et al^[33]^ reported that increased Her3 expression increased gefitinib resistance in patients with poorly differentiated CRC. Data from patients with KRAS wild-type mCRC in the GALGB/SWOG 80405 clinical trials revealed that patients with the primary tumor site of mCRC in the left colon could benefit from cetuximab.^[34]^ Her3 activation is responsible for cetuximab resistance developed under the pressure of the EGFR blockade,^[35]^ which may reduce cetuximab efficacy in patients with CRC.^[36]^ Kawakami et al^[37]^ indicated that high Her3 expression is an essential factor in cetuximab resistance and suggested that high expression of Her3 is associated with cetuximab treatment failure. On the basis of previous studies, our subgroup analysis revealed that patients with Her3 overexpression had a worse survival and tumor response after cetuximab treatment or cetuximab-based chemotherapy in mCRC.

Despite several studies reporting the association between Her3 expression and solid tumors, no similar meta-analysis investigated the benefits and risks of Her3 overexpression and CRC. As our study can provide a reference for the prognosis of CRC and a potential therapeutic target for CRC, the prognosis of patients with Her3 overexpression in different TNM stages warrants comprehensive studies. Furthermore, this study may pave a new path for the efficacy of cetuximab in the treatment of CRC. Our results suggested that considering the cost–benefit ratio of the therapy, Her3 expression should be tested before cetuximab treatment.

## 5 Limitations

There are several limitations in this study. First, all the data came from published articles and original data and individual patient data were unavailable, which implies that the results could be less precise. Second, HRs and 95% CIs in 3 articles were not provided directly, due to which we used software to extract the data, which may have reduced the accuracy and reliability of our results due to deviation. Besides, the high heterogeneity in our study may be attributed to the trial design and various therapeutic regimens. Finally, our study had a small sample size.

## 6 Conclusion

This meta-analysis suggested that Her3 overexpression is related to the clinical pathology and prognosis of CRC. Furthermore, we anticipate that the relationship between Her3 overexpression and prognosis is related to the TNM tumor stage. Her3 overexpression might be responsible for the nonefficacy of cetuximab treatment in patients with CRC. Owing to the limitations of the existing studies, further studies on the association between Her3 overexpression and the clinical pathology and prognosis of CRC are required. We are also looking forward to including further studies that analyzed the correlation between different TNM stages and prognosis in patients with a high Her3 expression.

## Author contributions

**Data curation:** Kaibo Guo, Guan Feng.

**Formal analysis:** Kai Zhang.

**Funding acquisition:** Minhe Shen, Shanming Ruan.

**Methodology:** Feiyu Shan.

**Resources:** Fengfei Shen.

**Software:** Leitao Sun.

**Supervision:** Shanming Ruan.

**Writing – original draft:** Qingying Yan, Kaibo Guo.

**Writing – review & editing:** Shanming Ruan.

Qingying Yan orcid: 0000-0002-0102-064X
